# Breath Metabolome Profiling Using Porous Carbon Material for Early Diagnosis of Laryngeal Cancer: Preliminary Results

**DOI:** 10.3390/cancers17213536

**Published:** 2025-11-01

**Authors:** Anna M. Kłeczek, Jadwiga Gabor, Jarosław Paluch, Robert Kwiatkowski, Jarosław Markowski, Katarzyna Mizia-Stec, Andrzej Małecki, Andrzej S. Swinarew

**Affiliations:** 1 Faculty of Science and Technology, University of Silesia, 41-500 Chorzów, Poland; 2 Department of Laryngology, Faculty of Medical Sciences in Katowice, Medical University of Silesia, 40-027 Katowice, Poland; 3 Radiotherapy Department, Katowice Oncological Center, 40-074 Katowice, Poland; 4 Frist Department and Clinic of Cardiology, Faculty of Medical Sciences in Katowice, Medical University of Silesia, 40-635 Katowice, Poland; 5 Department of Clinical Physiotherapy, The Jerzy Kukuczka Academy of Physical Education, 40-065 Katowice, Poland; 6 Institute of Sport Science, The Jerzy Kukuczka Academy of Physical Education, 40-065 Katowice, Poland

**Keywords:** laryngeal cancer, porous carbon material, exhaled air metabolome analysis, volatile organic compounds, non-invasive biomarkers, metabolomic signatures

## Abstract

**Background**: Early cancer detection remains a critical challenge in clinical oncology, requiring further development of innovative diagnostic methods with improved sensitivity and specificity. This study addresses the issue by investigating the potential of exhaled air metabolome analysis, using highly porous carbon material for sample collection, as a promising approach for the early diagnosis of laryngeal cancer. Volatile organic compounds (VOCs) present in exhaled breath are known to reflect underlying metabolic changes. This research explores the feasibility of using VOC-derived metabolomic signatures as non-invasive biomarkers for cancer detection. **Methods**: The primary objective was to evaluate exhaled air metabolome analysis as a diagnostic tool for individuals at risk of respiratory tract malignancies. The study involved 36 participants, including 13 patients diagnosed with laryngeal cancer and 23 healthy individuals serving as a control group. Breath samples were collected using a highly porous carbon material, selected for its superior sorption properties, enabling efficient capture and stabilization of VOCs. These samples were subsequently analyzed using gas chromatography-mass spectrometry (GC-MS) to identify and compare VOC patterns between the two groups. **Results**: Preliminary analysis revealed apparent differences in VOC profiles between cancer patients and healthy individuals, with cancer patients exhibiting elevated peak intensities for specific metabolites such as diethyl phthalate, nonadecane, and trimethyl-dodecane. Multivariate analysis using principal component analysis (PCA) demonstrated separation between the two groups, reflecting systematic differences in exhaled VOC signatures. **Conclusions**: This initial study supports the potential of breath VOC profiling for laryngeal cancer detection, laying the groundwork for further validation and refinement of this diagnostic approach. The use of porous carbon material facilitated efficient VOC capture, supporting its role in non-invasive breath analysis.

## Simple Summary

Early detection of laryngeal cancer is essential for effective treatment, yet current diagnostic methods are often invasive and uncomfortable. This study investigates a non-invasive approach based on the analysis of exhaled breath, which contains volatile compounds that may reflect disease-related metabolic changes. Breath samples were collected from individuals with and without laryngeal cancer using a specially designed porous carbon material that efficiently captures these compounds. The chemical profiles of the samples were then analyzed and compared. Preliminary results revealed apparent differences between the two groups. These findings provide a basis for further research into breath analysis as a supportive tool for early cancer diagnosis.

## 1. Introduction

Laryngeal cancer is a malignancy that affects the larynx—an organ crucial for breathing, phonation, and protecting the trachea from food aspiration. It is the second most common malignancy of the respiratory system, following lung cancer. According to the GLOBOCAN 2022 estimates [1], approximately 189,191 new cases of laryngeal cancer were diagnosed globally in 2022, with an age-standardized incidence rate (ASR) of 1.9 per 100,000 persons. The estimated 5-year prevalence reached 583,868 cases. Laryngeal cancer ranks as the 20th most commonly diagnosed cancer type. By 2030, the number of new cases is projected to rise to 227,685, further underscoring the need for effective diagnostic and prevention strategies [2]. It is significantly more common in males than females, with a male-to-female ratio of about 5:1, and is most frequently diagnosed in individuals over the age of 65 [3]. The most prevalent form of laryngeal cancer is squamous cell carcinoma, which constitutes approximately 95% of all cases [4] and most commonly originates in the glottic region, with less frequent involvement of the supraglottic and subglottic areas [5].

Despite advancements in medical research, the etiology of cancer remains complex and multifactorial. Within the spectrum of upper respiratory tract cancers, some cases remain idiopathic, where the specific causes are unknown. Genetic factors, unspecified environmental exposures, and inherent susceptibility contribute to the challenge of understanding these instances.

Examining established risk factors, particulate matter (PM) pollution, including PM2.5 and PM10, emerges as a significant environmental factor linked to increased incidence of upper respiratory tract cancer. PM originates from various sources, such as industrial emissions and vehicular exhaust. Its fine particles infiltrate the respiratory system, inducing chronic inflammation, DNA damage, and oxidative stress [6,7,8]. Prolonged exposure to PM2.5 is particularly associated with an elevated risk of upper respiratory tract cancers [9,10]. Larger particles like PM10 can also contribute to inflammatory responses, fostering cellular changes [11,12].

Alongside environmental factors, tobacco smoking remains a well-established and significant oncogenic factor. The complex chemical composition of tobacco smoke introduces various carcinogens, including polycyclic aromatic hydrocarbons and nitrosamines, directly damaging DNA and creating an environment conducive to tumor initiation and progression [13]. Smoking also induces chronic inflammation, suppresses the immune response, and compromises DNA repair mechanisms, further promoting the development of malignancies in the upper respiratory tract. Moreover, chronic and excessive alcohol consumption acts synergistically with smoking, and is associated with mucosal irritation and damage, impairs the body’s ability to metabolize carcinogens, and generates reactive oxygen species, further enhancing the risk of cancerous growth [14,15,16].

Eliminating all cancer risk factors is an impractical and unattainable goal due to the complex combination of genetic, environmental, and lifestyle factors. While adopting healthier habits can mitigate some risks, complete eradication is challenging. Therefore, effective cancer screening becomes crucial in the early detection and management of potential malignancies.

Laryngeal cancers present an especially formidable clinical challenge due to their typically asymptomatic progression in the early stages and the limitations of conventional screening methods. A notable number of patients diagnosed with respiratory tract cancers are identified at advanced stages, which significantly impacts their prognosis and treatment outcomes [17,18,19]. For instance, over 40% of all laryngeal cancer cases are detected at stages III and IV, indicating the disease’s spread to nearby tissues, lymph nodes, or distant organs [20]. Survival rates sharply decline for advanced stages compared to early-stage diagnoses, with a five-year survival rate of around 78% for early-stage laryngeal cancer, contrasting with only about 35% for advanced stages. This disparity underscores the critical role of early detection, which not only increases the likelihood of successful treatment but also enhances patients’ quality of life by potentially reducing the need for aggressive therapies and extensive surgical interventions.

Regular screenings enable the identification of abnormalities or precancerous conditions before they progress to advanced stages, allowing for timely intervention and improved treatment outcomes. Screening programs play a pivotal role in minimizing the impact of existing risk factors, enhancing the chances of successful cancer management, and ultimately improving overall public health.

Conventional screening modalities for upper respiratory tract cancers include physical examination with laryngoscopy and imaging techniques (X-ray, CT, MRI) for high-risk individuals. However, these approaches often lack sensitivity for very early or microscopic disease, suggesting that supplementary screening tools capable of detecting subtle biochemical or molecular signatures of malignancy could offer additional value. In this context, metabolomic analysis of exhaled breath has emerged as a promising avenue for non-invasive cancer detection.

Volatile organic compounds (VOCs) are a diverse group of organic chemicals that can easily transition into the gas phase. These compounds are produced in the body due to various metabolic processes and are exhaled through breath. In the context of cancer, VOCs can reflect metabolic alterations associated with malignancy, such as oxidative stress and lipid peroxidation. Analyzing VOCs in exhaled breath offers a promising, non-invasive approach to early cancer detection, potentially allowing for the identification of laryngeal cancer before clinical symptoms appear.

Selecting an appropriate method for collecting the exhaled breath phase is crucial, as it directly impacts the reliability and clinical utility of metabolomic analysis. Various VOCs present in exhaled breath could serve as potential biomarkers for the early detection of diseases [21,22], including lung and respiratory tract cancers [23,24,25]. However, the delicate nature of these compounds demands meticulous attention to the collection process to avoid degradation or loss of critical diagnostic information.

Gas sampling bags, though common for breath sample collection, have significant drawbacks [26,27,28]. They are prone to damage, risking sample integrity, and their bulkiness makes handling and transport difficult. Diagnostic compounds may degrade due to interactions with water vapor or the bag material, and manual transfer of samples can lead to contamination or loss, reducing analysis reliability. Additionally, material left on the bag walls often goes unanalyzed, potentially missing critical diagnostic information.

These limitations represent a notable handicap in studies necessitating precise results, particularly in areas like cancer diagnostics. They underscore the importance of adopting more reliable breath sample collection methods to ensure the required accuracy. Taking this into consideration, this study opted for a more precise approach by utilizing an organic sorbent—a highly porous carbon material based on a cyclic hexamer composed chiefly of four potassium glycidoxide molecules and two propylene oxide molecules [29].

Gas chromatography-mass spectrometry (GC-MS) was employed for the analysis of VOCs in exhaled breath. This technique enables the separation and precise identification of the individual compounds within a complex mixture, allowing for the development of detailed metabolic profiles.

This study aims to investigate the use of exhaled air metabolome analysis for the diagnosis of upper respiratory tract cancer, with the integration of porous carbon material for efficient sample collection. Similar studies have been conducted for asthma [30] and hypertension [31], revealing the potential of metabolomic analysis in providing non-invasive and sensitive diagnostic approaches for respiratory and cardiovascular conditions.

The following sections detail the methodology, findings, and implications of this study, underscoring the role of exhaled air metabolome analysis in advancing the diagnostic approach for laryngeal cancer.

## 2. Materials and Methods

The study comprised four main stages: selection of the sample collection method, material preparation, breath sample collection, and subsequent analysis using gas chromatography–mass spectrometry. Each step was designed to optimize the accuracy and reliability of detecting volatile organic compounds in exhaled breath for early-stage laryngeal cancer diagnosis.

### 2.1. Sample Collection Method

**Figure 1:**
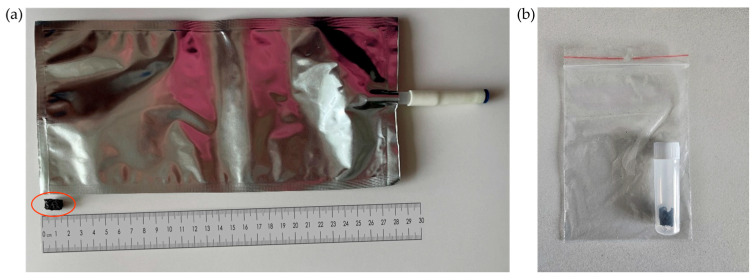
(**a**) Size comparison between porous carbon material (outlined in red) and a gas sampling bag; (**b**) collection vial.

The collection of exhaled breath using highly porous carbon material relies on both absorption and adsorption processes. Gaseous compounds permeate deeply into the material, while semi-volatile solids accumulate on the surface. The complex pore structure allows efficient trapping of breath constituents in a small, compact sorbent cartridge, providing a practical alternative to traditional large-volume breath collection bags (Figure 1).

Compared with other sampling approaches, the porous carbon sorbent offers several benefits. Adsorption on the activated carbon sorbent enables efficient pre-concentration of volatile and semi-volatile organic compounds and minimizes both dilution and analyte loss. Unlike polymer bags, which are prone to leakage, wall adsorption, and diffusion of contaminants, the porous carbon maintains VOC stability over extended storage periods. Moreover, because participants exhale directly through the cartridge, dead space is minimized and environmental background effectively excluded, ensuring that the analyzed compounds predominantly reflect endogenous metabolic products rather than external contaminants. This method also eliminates the need for bulky bags or external pumps, making the collection procedure short, patient-friendly, and suitable for clinical practice.

Other advantages include resilience to mechanical damage during transport, a straightforward operating principle (no auxiliary equipment required), and the potential for long-term sample storage under cooled conditions. In addition, the sorbent facilitates automated thermal desorption of compounds into the gas phase, minimizing analyte loss during preparation for chromatographic analysis. These combined features significantly enhance the efficiency and reproducibility of breath sampling.

### 2.2. Porous Material Preparation

**Figure 2:**
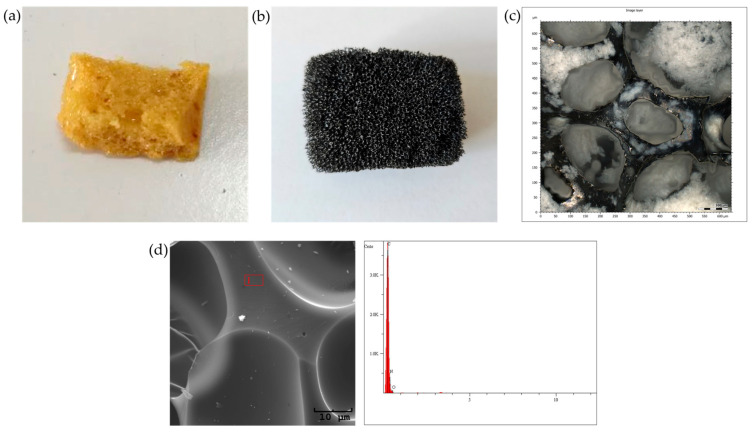
Porous material: (**a**) polyurethane base; (**b**) material after carbonization; (**c**) confocal microscopy image of the final material; (**d**) EDS elemental composition of the material after carbonization.

The porous material was synthesized following the methodology outlined in the publication [8], which involved the formation of a porous polyurethane base (Figure 2a). The resulting sorbent was cut into small cuboid pieces, approximately 2 × 1 × 1 cm in size, and was subsequently subjected to a two-stage carbonization process, consisting of crosslinking at 250 °C, followed by a high-temperature treatment at 950 °C. This process ensured the dehydration of the material and resulted in a highly porous material (Figure 2b,c) with a high carbon content (Figure 2d). The high porosity of the sorbent facilitates the effective penetration of both volatile and semi-volatile compounds from the exhaled breath during controlled exhalation.

The prepared carbon material was then placed into disposable plastic vials with perforated bottoms to ensure adequate airflow. Each tube was sterilized, labeled, and tightly sealed before use.

### 2.3. Sample Collection

The study was conducted on a cohort of 36 volunteers: 13 adult patients with diagnosed, biopsy-confirmed laryngeal carcinoma (various stages), and 23 healthy control individuals with no history of malignancy. The control and cancer groups were not matched for demographic or clinical variables such as age, sex, smoking status, or comorbidities. As this was a pilot investigation, the modest cohort size reflects the study’s exploratory nature, aimed at assessing the feasibility of the sampling and analytical approach and generating preliminary comparative data.

The study followed a uniform protocol for all subjects and was conducted in accordance with institutional and national ethical guidelines. After receiving standardized instructions, each participant exhaled freely into the test vial for a minimum of 3 s, and this process was repeated 10 times. The optimal number of exhalations required to sufficiently saturate the sorbent with compounds from the exhaled breath was determined empirically [6]. The procedure did not cause an exacerbation of respiratory symptoms in any of the participants. Collecting a single sample took approximately 5 min. The expiratory phase was collected consistently and without interference for all participants.

Immediately after sampling, the saturated sorbent was secured for transport. Each vial received proper labeling and was placed in a clean string bag. To guarantee stability during transit, the samples were stored in a portable refrigerator, where the temperature was kept below 0 °C. They were transported shortly after collection to the Institute of Materials Engineering at the University of Silesia, where the composition of exhaled air was analyzed using gas chromatography.

### 2.4. Gas Chromatography Analysis

Upon arrival at the laboratory, the sorbent material was transferred into gas-tight glass vials compatible with the Shimadzu GCMS-QP2010 chromatograph (Shimadzu, Kyoto, Japan). The vials were then loaded into the Shimadzu AOC-5000 Plus PAL (Shimadzu, Kyoto, Japan) autosampler and subjected to a thermodesorption process at 37 °C for 30 min, facilitating the release of volatile organic compounds for subsequent analysis.

The resulting headspace phase was subjected to chromatographic analysis using the Shimadzu GCMS-QP2010 Plus with a ZB 5MSI column. The temperature program was configured, starting at 36 °C (maintained for 1 min), then gradually increasing at a rate of 8 °C per minute until reaching 250 °C, where it was held for 25 min. The obtained data were examined by evaluating the characteristic features of the signals and the associated mass spectra.

### 2.5. Statistical Analysis

GC–MS peak intensities were obtained as absolute and relative values, with relative intensities scaled to the maximum peak per sample to allow semi-quantitative comparison. Baseline correction was applied to remove background signal contributions before data analysis. To show representative metabolomic profiles for each group, averaged chromatograms were generated by aligning retention times and averaging corresponding signal intensities across samples within each cohort. For multivariate analysis, a GC–MS intensity matrix was constructed by grouping retention times between 0 and 50 min into uniform 0.1 min bins, enabling alignment of chromatographic profiles and minimizing retention time variability between runs. The resulting binned intensities were standardized (zero mean, unit variance) to ensure comparability across samples. Principal Component Analysis (PCA) was then applied to reduce data dimensionality, summarize the variance structure of VOC profiles, and explore separation trends between laryngeal cancer patients and healthy controls. No formal hypothesis testing was performed, as this pilot study focused on evaluating the feasibility and reproducibility of the analytical workflow.

## 3. Results

A preliminary analysis of GC-MS data was conducted to elucidate volatile metabolomic signatures associated with laryngeal carcinoma. The primary objective was the qualitative and semi-quantitative characterization of VOCs present in exhaled breath, with an emphasis on exploring disease-specific metabolic alterations.

Breath condensate samples were obtained from a cohort of 36 individuals, comprising histopathologically confirmed laryngeal cancer patients and healthy controls. The acquired chromatographic data were carefully evaluated, with particular attention given to high-intensity peaks exhibiting consistent retention times and unique mass spectral fragmentation patterns.

Comparative chromatographic profiles revealed marked differences in VOC expression between oncological and control samples. Breath samples from laryngeal cancer patients exhibited significantly elevated signal intensities for several compounds, showing consistent patterns across multiple samples. These signals were either absent or considerably less pronounced in chromatograms from healthy individuals.

**Figure 3:**
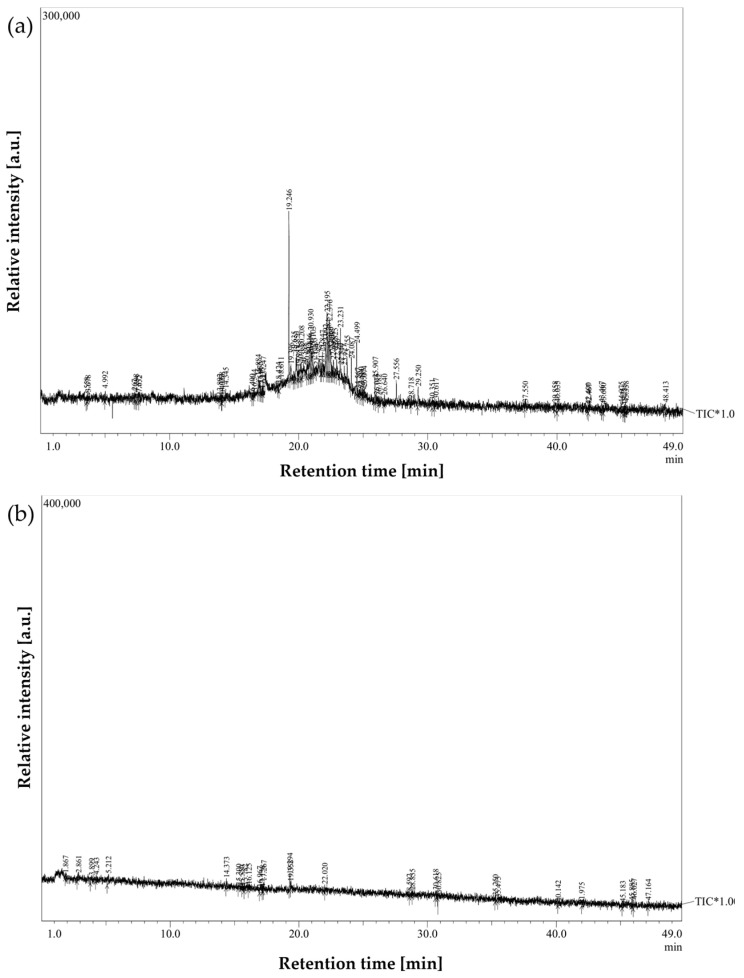
Breath sample chromatograms from: (**a**) a laryngeal cancer patient; (**b**) a healthy participant from the control group.

**Figure 4:**
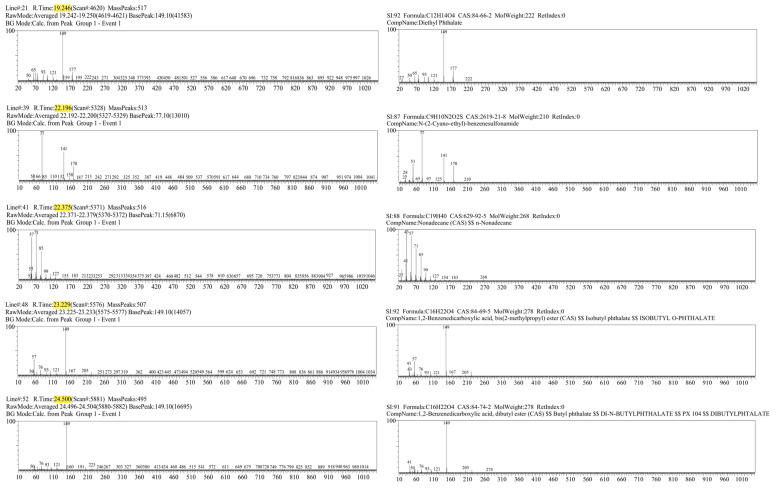
Mass spectra of the 5 most characteristic peaks from the laryngeal cancer patient sample (**left**), with corresponding compound identification using the Wiley library (**right**).

Figure 3 and Figure 4 illustrate representative chromatograms and peak deconvolution from a single cancer patient and a matched healthy control, exemplifying the differences observed throughout the cohort.

**Figure 5:**
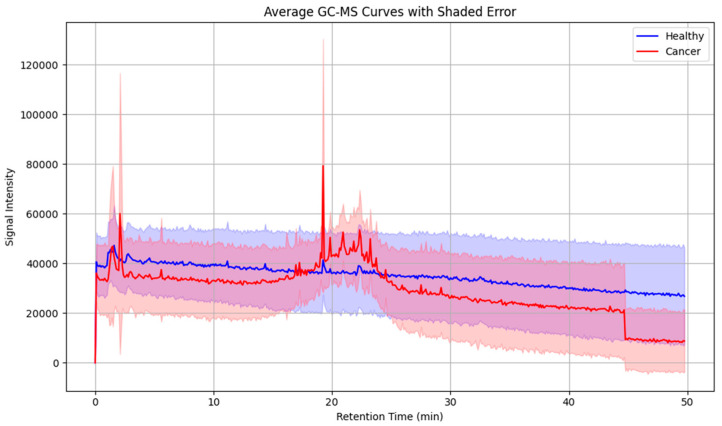
Averaged GC-MS chromatograms of exhaled breath samples from laryngeal cancer patients and healthy controls, illustrating distinctive volatile organic compound profiles associated with disease status. Shaded areas indicate within-group variability.

To further assess intergroup variability, average chromatograms were generated for all cancer patients and healthy controls (Figure 5). These averaged profiles highlighted a consistent set of peaks upregulated in the cancer group, particularly within the mid- to late-retention time range, thereby reducing individual variability and underscoring recurrent VOC features.

As no external calibration gases or internal standards were employed during GC-MS acquisition, the analysis remained semi-quantitative and focused on relative differences in signal intensity rather than absolute VOC concentrations. Relative differences in peak areas were interpreted as indicative of relative differences in VOC concentrations, based on the consistent sampling protocol used across participants, which involved multiple exhalations.

**Figure 6:**
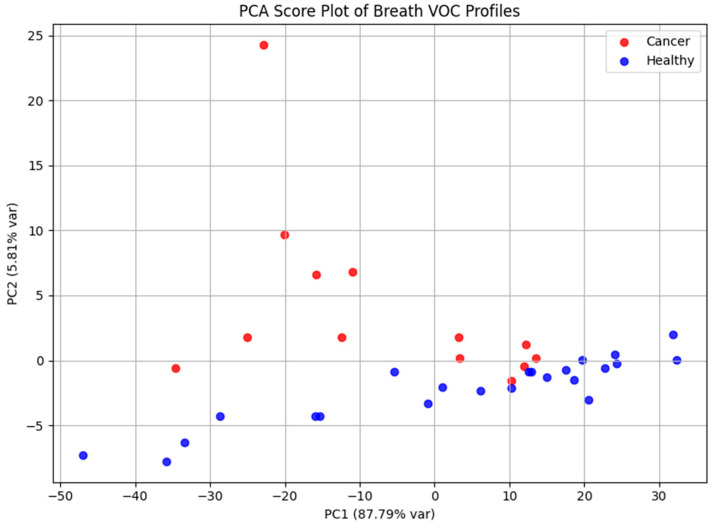
PCA score plot based on GC-MS data, illustrating separation between laryngeal cancer patients and healthy controls according to their volatile metabolomic signatures. The distribution of samples reflects systematic differences in exhaled VOC profiles between the two groups.

Principal Component Analysis was applied to explore the dataset’s structure and evaluate its capacity to discriminate between cohorts (Figure 6). The PCA score plot demonstrated apparent separation between laryngeal cancer patients and healthy controls, reflecting systematic differences in exhaled VOC profiles. PC1 and PC2 accounted for 87.79% and 5.81% of the total variance, respectively, highlighting that the majority of variance in the dataset is captured by the first principal component.

For each participant diagnosed with laryngeal cancer, five most distinctive peaks were identified based on their retention times (Table 1). The compounds corresponding to these peaks were determined by calculating the similarity index (SI) and referencing the Wiley mass spectral library for accurate identification. The similarity index represents the percentage similarity between the obtained mass spectrum and the reference spectrum in the library, with higher values indicating a closer match. In this study, tentative identifications were accepted for SI ≥ 75%, while compounds with lower scores were classified as uncertain and interpreted with caution. It is important to note, however, that low SI values do not necessarily imply the absence of chemically meaningful information. In breath metabolomics, reduced match quality may arise from several factors, including the limited scope of spectral libraries that may not contain all possible endogenous or exogenous VOCs, low analyte concentrations near the detection threshold, which can distort spectral features, and partial co-elution of compounds within complex breath matrices. Consequently, even spectra with modest similarity indices may reflect genuine chemical signals, particularly when they appear consistently across multiple samples or within biologically relevant retention time windows.

## 4. Discussion

The discernible disparities observed in the volatile organic compound profiles between patients with laryngeal carcinoma and healthy controls underscore the presence of cancer-associated metabolic perturbations. The breath analysis methodology employed in this study—based on the use of a highly porous carbon sorbent—proved effective in capturing these metabolomic differences with notable resolution and reproducibility. The analytical outcomes demonstrated substantial inter-sample variability coupled with high chromatographic resolution, enabling stratification of participants based on disease status. Distinct peak patterns in the chromatograms reflect the analytical sensitivity of this approach in detecting subtle biochemical deviations.

The proposed technique—molecular analysis of the exhaled breath phase following adsorption on porous activated carbon with subsequent thermal desorption and GC–MS profiling—offers significant advantages. Currently, no standardized protocol exists for breath sample collection, which introduces variability and raises concerns regarding reproducibility and specificity of results. Adsorption on activated carbon provides highly efficient pre-concentration of volatile and semi-volatile organic compounds, while simultaneously minimizing dilution and loss of analytes. More importantly, this approach enables selective trapping of endogenous metabolites and allows subsequent desorption under controlled conditions, thereby reducing the risk of interference from transient environmental contaminants. As a result, the method increases analytical specificity and ensures that the measured molecular signatures can be more reliably attributed to the metabolic activity of laryngeal tumor cells rather than exogenous sources.

Preliminary compound identification revealed the recurrent presence of a particular class of chemicals—phthalates—in breath samples from cancer patients. Phthalates, a group of diesters derived from phthalic acid, are ubiquitous in consumer and industrial products, serving primarily as plasticizers in polymers. These compounds are often detected in aerosolized form, especially in enclosed or poorly ventilated environments. Given their exogenous nature—being neither synthesized endogenously nor physiologically necessary—their detection in exhaled breath is most plausibly attributed to environmental exposure via inhalation or dermal absorption, or to downstream metabolites of more complex phthalate derivatives introduced into the body.

Among the compounds identified through GC-MS analysis, diethyl phthalate (DEP) was detected in a substantial number of exhaled breath samples from patients diagnosed with laryngeal cancer. DEP is a widely utilized synthetic compound, primarily employed as a plasticizer in the production of polymers and found in a broad range of consumer and industrial products, including cosmetics, personal care items, medical devices, and packaging materials. Consequently, individuals are routinely exposed to DEP through various environmental and occupational channels. One of the primary routes of systemic uptake is inhalation of indoor air contaminants, especially in enclosed environments where phthalates may be released from building materials, furniture, or plastic-containing consumer goods. Additionally, dermal absorption of DEP from personal care products represents a significant exposure pathway, with studies demonstrating measurable systemic levels of phthalates following regular use of lotions, deodorants, or perfumes [32,33]. Once absorbed, DEP undergoes partial metabolism in the liver and may circulate as either the parent compound or its metabolites, some of which possess sufficient volatility to be eliminated via exhaled breath. Importantly, in the oncological context, the altered metabolic landscape of cancer patients may further contribute to the systemic accumulation or modified excretion of xenobiotics such as DEP. Aberrant activity of detoxification enzymes, oxidative stress pathways, and cytochrome P450 systems, often seen in malignancies, may enhance the persistence of phthalates or their transformation into volatile organic derivatives. This could explain the observed increase in signal intensity for DEP in breath samples from laryngeal cancer patients, as compared to healthy controls. Moreover, the detection of DEP across multiple cancer patient profiles supports the hypothesis that its presence is not incidental or solely reflective of ambient contamination, but rather indicative of consistent exposure patterns or pathophysiological alterations in phthalate metabolism. Therefore, while DEP is an exogenous compound not endogenously synthesized by human cells, its appearance in breath samples—independent of any potential leaching from the sorbent material—may reflect a combination of environmental exposure, personal habits, and disease-specific metabolic dysregulation. This strengthens its relevance as a potential candidate for inclusion in VOC-based biomarker panels for non-invasive cancer diagnostics.

In addition to phthalates, the analysis revealed elevated levels of alkanes and branched hydrocarbons—such as nonadecane and trimethyl-dodecane—in the exhaled breath of patients with laryngeal cancer. Alkanes have previously been reported as potential biomarkers for various cancers and inflammatory conditions, primarily due to their origin in lipid peroxidation processes [21]. In malignancy, excessive production of reactive oxygen species can induce oxidative degradation of polyunsaturated fatty acids in cellular membranes, leading to the generation of volatile alkanes such as ethane, pentane, and higher hydrocarbons. These compounds may diffuse into the bloodstream and be eliminated via exhalation. The presence of heavier alkanes, including nonadecane, may indicate more complex oxidative mechanisms or microbiome-associated metabolism. Elevated breath alkane levels have been documented in head and neck cancers [25,34], and the current findings are consistent with those observations, suggesting a distinct hydrocarbon profile in laryngeal cancer that likely reflects tumor-associated oxidative stress and membrane lipid degradation.

The comprehensive identification and validation of candidate biomarkers are critical to elucidating their predictive value in the context of laryngeal cancer. Breath-based metabolomic profiling presents a promising frontier for the early detection, molecular phenotyping, and longitudinal monitoring of malignancies. By reflecting the biochemical landscape of systemic pathophysiological changes, metabolomics offers a powerful lens through which oncogenesis can be detected and characterized at a molecular level. Specifically, this analytical strategy holds potential to provide clinically actionable insights that may guide personalized therapeutic interventions.

To enhance diagnostic precision and clinical relevance, future investigations must be conducted on larger, statistically powered cohorts. Expanding the sample size will facilitate robust biomarker validation, enable the characterization of disease-specific metabolomic fingerprints, and support stratification across tumor stages.

Comparative analysis of breath metabolomes between healthy individuals and patients at various disease stages may enable the delineation of temporal biomarker patterns indicative of tumor progression, treatment response, or relapse risk. An ideal biomarker panel should exhibit high sensitivity and specificity, be stage-discriminative, and possess the capacity to inform on therapeutic responsiveness. Establishing a detailed biomarker panel for laryngeal carcinoma could enable clinicians to tailor interventions based on individual molecular profiles, minimizing reliance on empirical treatment approaches. This, in turn, could lead to improved clinical outcomes, reduced treatment-related morbidity, and more efficient resource utilization.

However, the modest sample size of this pilot study and the absence of demographic and clinical matching between case and control groups—factors known to influence the composition of exhaled VOCs—inevitably limit statistical power and generalizability. In addition, exogenous influences such as dietary habits, recent food intake, and environmental exposures may contribute to interindividual variability in VOC profiles. Although participants were instructed to refrain from eating, drinking, and smoking prior to sample collection, residual diet-related volatiles (e.g., alcohols, aldehydes, terpenes) cannot be fully excluded and may have acted as confounding factors. Consequently, the observed metabolomic trends should be interpreted as preliminary, reflecting the feasibility of the methodology rather than definitive disease-specific signatures.

To ensure translational viability, further research is imperative to refine analytical methodologies, validate candidate biomarkers across diverse populations, and confirm the reproducibility of results under clinical conditions. In future studies, the use of calibrated gas standards during the GC–MS analysis phase will enable the quantification of absolute compound concentrations, allowing more precise comparisons of peak intensities across samples. Only through rigorous, large-scale validation can breath-based metabolomics be effectively integrated into standard oncological diagnostic workflows.

Successful clinical implementation, however, requires consideration of additional practical factors. Logistically, the use of compact porous carbon sorbent cartridges enables rapid, patient-friendly sample collection without specialized equipment, facilitating potential adoption in outpatient or primary care settings. From a regulatory perspective, breath-based diagnostic workflows must comply with medical device standards and obtain approvals from relevant health authorities to ensure safety, reproducibility, and data integrity. Economically, this approach offers potential cost advantages over invasive procedures due to simple, minimally labor-intensive sample collection; nevertheless, investment in standardized GC–MS instrumentation, staff training, and quality control is necessary to maintain consistent performance. Addressing these operational, regulatory, and financial considerations is essential for translating preliminary findings into clinically actionable diagnostic tools.

## 5. Conclusions

The preliminary findings suggest that VOC profiling via GC-MS holds substantial promise for the development of breath-based diagnostic platforms in oncological screening. The implementation of porous carbon-based materials for exhaled breath sampling has demonstrated notable analytical efficacy, enabling the efficient capture and stabilization of a diverse spectrum of volatile organic compounds with high sensitivity and reproducibility. The construction of comprehensive metabolomic profiles, coupled with the identification of disease-specific volatile biomarkers, underscores the considerable potential of breath analysis as a non-invasive diagnostic and monitoring tool in the context of laryngeal cancer.

To facilitate clinical translation, sustained research efforts are imperative—particularly those aimed at standardizing the methodology, validating analytical reproducibility, and establishing robust biomarker panels. Future studies should prioritize the recruitment of larger, well-characterized patient cohorts and focus on the precise identification of VOCs associated with malignancies of the upper respiratory tract, with an emphasis on laryngeal squamous cell carcinoma. Moreover, the integration of advanced computational approaches, including artificial intelligence, machine learning algorithms, and multivariate statistical modeling, is expected to significantly enhance diagnostic precision, pattern recognition, and classification capabilities. Such interdisciplinary advancements will be instrumental in refining breathomics as a viable modality for early cancer detection, disease stratification, and personalized therapeutic decision-making in oncological practice.

## Figures and Tables

**Table 1:** Potential chemical compounds identified in the exhaled breath of patients diagnosed with laryngeal cancer.

Sample ID	Identified Chemical Compound	Retention Time [s]	SI Index [%]
1	Diethyl phthalate	19.246	92
	N-(2-Cyano-ethyl)-benzenesulfonamide	22.196	87
	Nonadecane	22.375	88
	1,2-Benzenedicarboxylic acid, bis(2-methylpropyl) ester	23.229	92
	Dibutyl phthalate	24.500	91
2	1-Hexanol, 5-methyl-2-(1-methylethyl)-	20.796	88
	Dibutyl phthalate	24.479	90
	Hexadecane, 2,6,10,14-tetramethyl-	22.313	88
	1,2-Benzenedicarboxylic acid, bis(2-methylpropyl) ester	23.192	89
	Dodecane, 4-methyl-	20.908	87
3	2-(2′,4′,4′,6′,6′,8′,8′-Heptamethyltetrasiloxan-2′-yloxy)-2,4,4,6,6,8,8,10,10-nonamethylcyclopentasiloxane	19.825	76
	Silicate anion tetramer	22.071	73
	Tetradecane	22.342	81
	1,2-Benzenedicarboxylic acid, bis(2-methylpropyl) ester	23.192	83
	Dodecane, 2,6,10-trimethyl-	20.900	82
4	Diethyl phthalate	19.217	93
	Dibutyl phthalate	24.467	96
	Benzenesulfonamide, N-butyl-	22.158	90
	Octasiloxane, 1,1,3,3,5,5,7,7,9,9,11,11,13,13,15,15-hexadecamethyl-	24.054	80
	1,2-Benzenedicarboxylic acid, bis(2-methylpropyl) ester	23.196	93
5	Diethyl phthalate	19.229	94
	Phthalic acid, butyl undecyl ester	24.483	82
	Benzenesulfonamide, N-butyl-	22.171	89
	1,2-Benzenedicarboxylic acid, bis(2-methylpropyl) ester	23.208	93
	Nonadecane	20.917	85
6	Diethyl phthalate	19.242	86
	Dodecane, 2,6,10-trimethyl-	20.917	88
	1,2-Benzenedicarboxylic acid, bis(2-methylpropyl) ester	23.217	85
	Dodecane, 2,6,11-trimethyl-	22.367	86
	Dibutyl phthalate	24.488	85
7	Benzenesulfonamide, N-butyl-	22.175	90
	2-(2′,4′,4′,6′,6′,8′,8′-Heptamethyltetrasiloxan-2′-yloxy)-2,4,4,6,6,8,8,10,10-nonamethylcyclopentasiloxane	19.838	78
	Silicate anion tetramer	22.096	79
	Dibutyl phthalate	24.488	93
	1,2-Benzenedicarboxylic acid, bis(2-methylpropyl) ester	23.213	88
8	Dodecane, 2,6,10-trimethyl-	20.938	87
	Nonadecane	20.783	86
	1,2-Benzenedicarboxylic acid, bis(2-methylpropyl) ester	23.238	84
	Dibutyl phthalate	24.513	87
	Dodecane, 2,6,10-trimethyl-	22.392	87
9	Diethyl phthalate	19.221	95
	Cycloheptasiloxane, tetradecamethyl-	17.225	86
	Dimethyl phthalate	16.950	89
	Cyclooctasiloxane, hexadecamethyl-	19.821	80
	Cyclohexanecarboxylic acid, 1-phenyl-, methyl ester	30.958	76
10	Diethyl phthalate	16.210	91
	Octasiloxane, 1,1,3,3,5,5,7,7,9,9,11,11,13,13,15,15-hexadecamethyl-	26.170	77
	Cyclodecasiloxane, eicosamethyl-	28.295	71
	1,1,1,3,5,7,7,7-Octamethyl-3,5-bis(trimethylsiloxy)tetrasiloxane	16.805	61
	Benzene, (2-nitropropyl)-	27.920	64
11	1,2-Butadiene	1.245	87
	2-Butyne	1.485	85
	Diethyl Phthalate	16.205	86
	(2,3-Diphenylcyclopropyl)methyl phenyl sulfoxide, trans-	27.915	56
	Silicate anion tetramer	21.03	40
12	Furan, tetrahydro-	2.133	83
	Cyclohexene, 4-ethenyl-	1.288	34
	Xanthine	1.792	26
	2H-1,3-Benzoxazine, octahydro-2-(3-nitrophenyl)-, cis-	6.992	24
	N,N-Diethyl-1,1,1-trimethylsilylamine	14.325	24
13	1,1,1,2,2-Pentafluoropropane	1.465	78
	1,2,4-Oxadiazole, 3,5-di(4-nitrophenyl)-	23.675	21
	Benzo[b]thiophene, 2-ethyl-5,7-dimethyl-	30.185	20
	Benzeneacetic acid, 3-methoxy-4-[(trimethylsilyl)oxy]-, ethyl ester	32.855	20
	2-[(4-Nitrophenyl)sulfanyl]-4-(trichloromethyl)pyrimidine	39.555	24

## Data Availability

Research data are stored in an institutional repository and will be shared upon request to the corresponding author.
